# Low-Dose Metformin as a Monotherapy Does Not Reduce Non-Small-Cell Lung Cancer Tumor Burden in Mice

**DOI:** 10.3390/biomedicines9111685

**Published:** 2021-11-14

**Authors:** Nicole L. Stott Bond, Didier Dréau, Ian Marriott, Jeanette M. Bennett, Michael J. Turner, Susan T. Arthur, Joseph S. Marino

**Affiliations:** 1 Distance Education, Technology and Integration, University of North Georgia, Dahlonega, GA 30597, USA; nicole.bond@ung.edu; 2 Laboratory of Systems Physiology, Department of Applied Physiology, Health, and Clinical Sciences, University of North Carolina at Charlotte, Charlotte, NC 28223, USA; miturner@uncc.edu (M.J.T.); sarthur8@uncc.edu (S.T.A.); 3 Department of Biological Sciences, University of North Carolina at Charlotte, Charlotte, NC 28223, USA; ddreau@uncc.edu (D.D.); imarriot@uncc.edu (I.M.); 4 Department of Psychological Science, University of North Carolina at Charlotte, Charlotte, NC 28223, USA; jbenne70@uncc.edu

**Keywords:** Lewis lung model, lung cancer, skeletal muscle, cachexia

## Abstract

Non-small-cell lung cancer (NSCLC) makes up 80–85% of lung cancer diagnoses. Lung cancer patients undergo surgical procedures, chemotherapy, and/or radiation. Chemotherapy and radiation can induce deleterious systemic side effects, particularly within skeletal muscle. To determine whether metformin reduces NSCLC tumor burden while maintaining skeletal muscle health, C57BL/6J mice were injected with Lewis lung cancer (LL/2), containing a bioluminescent reporter for in vivo tracking, into the left lung. Control and metformin (250 mg/kg) groups received treatments twice weekly. Skeletal muscle was analyzed for changes in genes and proteins related to inflammation, muscle mass, and metabolism. The LL/2 model effectively mimics lung cancer growth and tumor burden. The in vivo data indicate that metformin as administered was not associated with significant improvement in tumor burden in this immunocompetent NSCLC model. Additionally, metformin was not associated with significant changes in key tumor cell division and inflammation markers, or improved skeletal muscle health. Metformin treatment, while exhibiting anti-neoplastic characteristics in many cancers, appears not to be an appropriate monotherapy for NSCLC tumor growth in vivo. Future studies should pursue co-treatment modalities, with metformin as a potentially supportive drug rather than a monotherapy to mitigate cancer progression.

## 1. Introduction

Lung cancer is the second most common cancer and represents ~13% of all new cancer cases in the United States (SEER, National Cancer Institute). Lung cancer contributed to ~145,000 fatalities in 2019 [1], with the yearly diagnoses expected to reach 225,000 in 2030, in the United States alone [2]. Cigarette smoke is one of the largest contributors to lung cancer diagnoses, but now it has now been established that a combination of lifestyle, genetic, and environmental components contributes to an individual’s risk and development of lung cancer [3]. Specifically, factors that put individuals at a greater risk for lung cancer include cigarette smoke, environmental pollutants, alcohol consumption, adverse dietary consideration, physical inactivity, and hereditary markers [3]. Lung cancer patients have a 5 year relative survival rate of only 19% (16% for men and 22% for women), making it one of the lower survival rates among cancers [1]. While treatments continue to improve, the prevalence and severity of lung cancer necessitates more refinement of treatment modalities.

Continuous advances are bringing new insight into oncology therapeutics [4], especially through drug repositioning [5]. This is an attractive tactic since new drug characterization and approval requires an extensive investment in time and money [6]. Observational studies, pre-clinical trials, and clinical trials have provided insights into the efficacy of drug repositioning for cancer prevention and cancer therapy [7]. 

Metformin canonically facilitates improved insulin sensitivity and overall glucose uptake for type 2 diabetes (T2D) patients, but recent studies show the potential of repositioning metformin due to its anti-cancer properties [8,9,10,11,12]. Importantly, the literature suggests that metformin decreases lung cancer risk for T2D patients and increases survival for lung cancer patients with co-morbid T2D [13,14,15,16]. Whether this is due to normalization of glycemia and insulinemia, or results from a direct effect on tumor burden, remains to be determined.

Metformin elicits anti-tumorigenic effects in many cancers, including prostate, colon, skin, and obesity-activated thyroid cancer [10,12,17,18]. In cancers, many signaling pathways components, including AMPK, mTOR, MAPK, and insulin-like growth factors contribute to the anti-tumorigenic effects of metformin [19]. In particular, metformin activates AMPK inhibiting cell mitosis and proliferation, particularly via protein p53 activation [5]. While metformin demonstrates anti-neoplastic effects via cell cycle arrest, the efficacy of metformin and the mechanism underlying this agent’s action on non-small-cell lung cancer (NSCLC) tumor development remains unclear. Filling this knowledge gap is crucial to the successful repositioning of metformin as an anti-cancer therapeutic. Utilizing metformin independently or in conjugation with other treatment modalities could mitigate the side effects many cancer patients experience while receiving more potent oncology therapeutics.

Following diagnosis, lung cancer patients often undergo surgical procedures, chemotherapy, or radiation, but these can drive systemic complications, negatively affecting patient welfare and recovery timelines. One of the most common systemic effects of conventional cancer treatment is cachexia, the rapid loss of skeletal muscle and adipose tissue [20,21]. Cachexia occurs in more than 50% of lung cancer patients undergoing chemotherapy, radiotherapy, or a combination of both [22,23], and more than 60% of patients with advanced NSCLC present respiratory complications and increased rates of cachexia [24]. Furthermore, patients with cancer-induced cachexia often exhibit a lower tolerance and responsiveness to chemotherapy, shortened survival times, far greater symptom burdens, and systemic inflammation [25,26]. Higher morbidity and mortality rates also correlate with the degree of weight loss and rapid decreases in BMI, both of which are independent prognostic factors for cancer patients, with or without cachexia [24,27].

Few treatment options are available for cachexia and these effects are irreversible even during remission, making such repercussions even more debilitating [28,29]. Metformin may be an attractive target to manage cancer-induced metabolic dysfunction and cachexia. Within skeletal muscle, which is the largest insulin-sensitive tissue in the body, metformin increases peroxisome proliferator-activated receptor-coactivator-1α (PGC-1α) protein expression, a transcriptional co-activator involved in mitochondrial biogenesis, glucose metabolism, and muscle fiber type differentiation [30]. PGC-1α increases the expression of genes involved in energy metabolism, which is thought to protect skeletal muscle from atrophy, and suppresses forkhead box O3 (FoxO3), a transcription factor that induces the expression of ubiquitin-ligases involved in atrophy [31]. Metformin also preserves the satellite cell pool in a lower metabolic state which sustains quiescence and delays satellite cell activation [32]. Maintenance of the stem cell population is crucial for preservation of skeletal muscle mass, repair, and function [33].

Although metformin has been used as an anti-cancer therapy in clinical trials, its efficacy against NSCLC remains understudied. Furthermore, it is currently unknown how the combination of metformin treatment and NSCLC directly influences skeletal muscle health and metabolism. In the present study, we have investigated whether metformin treatment suppresses tumor growth in C57BL/6J mice with NSCLC, and we have investigated the effects of NSCLC tumor progression on skeletal muscle health. Importantly, we have employed a mouse model in the present study where the animals are neither obese nor diabetic and this has allowed us to investigate the direct effects of metformin on tumor burden. Determining the efficacy of metformin therapy against NSCLC could provide new treatment options for cancer patients and provide valuable insights into the physiological disparities that underlie NSCLC progression.

## 2. Materials and Methods

### 2.1. Experimental Animals

**Figure 1:**
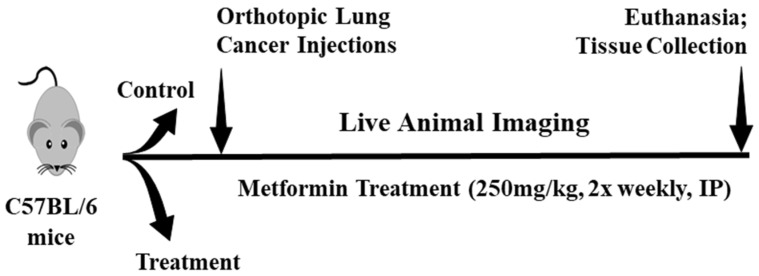
Experimental timeline for Lewis lung carcinoma development in an immunocompetent mouse model. Male (*n* = 12) and female (*n* = 12) C57BL/6J mice were implanted with 1000 Lewis lung carcinoma cells harboring luciferase reporter expression. Live animal imaging was continuous for the duration of the study. Once a bioluminescent signal was detected, vehicle or metformin treatment (250 mg/kg, 2× weekly, intraperitoneal injection) began.

Six-week-old male (*n* = 12) and female (*n* = 12) C57BL/6J mice (Jackson Laboratory, Bar Harbor, ME, USA) were randomly assigned (manually) into a control group (lung cancer without metformin treatment) (*n* = 12; 6 males, 6 females) and a metformin treatment group (lung cancer with metformin treatment) (*n* = 12; 6 males, 6 females). All animals were housed individually in cages with filter lids and placed in rooms with a 12:12 h light:dark cycle. Mice were housed in cages measuring 7.5 inches in width, 11.5 inches in length and 5 inches in height (Allentown Inc. and Ancare, Bellmore, NY, USA). The floor surface area was 86.25 square inches. Teklad corncob bedding was used throughout the study (7092A; Envigo, Cumberland, VA, USA). For enrichment, all cages included a small plastic hide (Bio-care, Flemington, NJ, USA) and a Nestlet 2 inch square for nestling (Ancare, Bellmore, NY, USA). The animal housing facility was equipped with 24-h temperature monitoring and alarms to ensure a constant ambient temperature of 65–75 °F and 20–60% humidity (depending on season). Animals were acclimated for 5 days prior to use. When an animal exhibited signs of distress (>20% reduction in body weight), the animal was immediately captured by daily weigh-ins and euthanized. Control (*n* = 7) and metformin (*n* = 9) animals completed the study and were used in statistical calculations. Some control mice (*n* = 5) and metformin-treated mice (*n* = 3) mice presented extreme tumor burdens and did not survive for the full length of study and were excluded from statistical calculations (Table 1). Figure 1 outlines the study progression.

All mice were provided with ad libitum access to water and standard rodent chow (Teklad Diets 2919; Envigo, Cumberland, VA, USA). Food mass was measured weekly and the total amount of food consumed over the study was used to determine total caloric intake. The energy density of the standard rodent chow was 3.3 kcal/g. Male and female C57BL/6J mice were used to address metformin’s efficacy on reducing lung tumor burden in immunocompetent mice. The Lewis lung carcinoma immunocompetent mouse model mimics lung tumor development including the immune system modulations. The non-small-cell lung carcinoma (NSCLC) Lewis lung carcinoma (LL/2) cells are syngeneic with C57BL/6J mice and stably and constitutively expresses a luciferase reporter (Imanis Life Sciences, Rochester, MN, USA), allowing tumor growth monitoring over time with a live animal imaging system. The LL/2 orthotopic model effectively mimics lung cancer growth and tumor burden in accordance with other murine Lewis lung cancer models [34,35,36]. All aspects of this study were approved by the Institutional Animal Care and Use Committee at The University of North Carolina at Charlotte.

### 2.2. Culturing Non-Small-Cell Lung Cancer Cells

NSCLC cells (Imanis Life Sciences, Rochester, MN, USA) were grown in standard growth media (Dulbecco’s Modified Eagle Medium) with 10% fetal bovine serum and 1% penicillin-streptomycin. Cells were passaged with 2 µg/mL puromycin to maintain high luciferase fluorescence expression. Cells were maintained at 37 °C for 48 h or until predetermined time points.

### 2.3. Orthotopic Injection

Animal hair was removed from the ventral and left thoracic regions and were then aseptically prepared. Prior to receiving an LL/2 cancer injection, all animals were imaged and baseline images acquired using an in vivo imaging system (IVIS). Under anesthesia (1–3% isoflurane), mice received one orthotopic lung injection of LL/2 cells into the left lung. LL/2 cells (1.0 × 10^3^) were administered in PBS and Matrigel^®^ (10 µg; Dulbecco’s Modified Eagle’s Medium with 50 ug/mL gentamycin phenol red free, Corning, Glendale, AZ, USA). Matrigel^®^ facilitated both tumor cell growth and homing within the lung tissue [36]. A small incision (3–5 mm) was made to expose the area surrounding the seventh and eighth ribs. Cells were injected orthotopically into the lung using a sterile 29-gauge syringe and the incision was closed with a wound clip. Following surgery, all mice were individually housed and allowed to recover for one week. Animal weights were recorded weekly throughout the study. Any mouse showing signs of distress or exceeding 20% body mass loss was euthanized in accordance with approved IACUC guidelines.

### 2.4. In Vivo Imaging

Tumor growth in all animals was initially monitored weekly using bioluminescent imaging via IVIS. Cell visualization in vivo occurred by giving all animals D-luciferin (150 mg/kg) 15 min prior to imaging. The area to be imaged was shaved and cleaned to remove any hair that could interfere with the bioluminescent signal detected. All images were captured within a 30 min window following D-luciferin injection. All mice were imaged weekly until a bioluminescent signal was detected. Following detection, each mouse was imagined bi-weekly and treatment commenced.

### 2.5. Metformin Treatment

Control and metformin-treated mice were injected intraperitoneally (i.p.) with saline (PBS, 1×) and metformin (250 mg/kg, twice weekly). Metformin hydrochloride (1084; Sigma Aldrich, St. Louis, MO, USA) was dissolved in 1× PBS and sterile filtered (0.2 µm) for a final dose of 250 mg/kg. Metformin preparations were cultured on nutrient agar plates to ensure sterility. This metformin dose is commonly used in many mouse cancer studies [37,38]. While metformin dosing is typically daily, the mice used in this study also received injections for bioluminescent imaging so we minimized administrations to twice weekly. Control mice received a placebo of 1× PBS solution via an i.p. injection twice a week. At 5 weeks post-tumor implantation, mice were euthanized (>4% isoflurane), and tissue was collected, snap frozen on liquid nitrogen, and stored at −80 °C.

### 2.6. Tumor Burden

Tumor burden was assessed with the Living Image analysis (Version 4.5.5, Perkin Elmer, USA). The region of interest (ROI) was determined by outlining the tumor bioluminescent signal with minimum detection parameters set to 5%. Brightness, contrast, and opacity were maintained between all images regardless of time point. A separate ROI was drawn on each mouse to determine background signal. Mice with metastases were identified as having more ROIs at a single time point. Each bioluminescent signal was first normalized to the background signal for the same image and all animals were normalized to the baseline image of the same mouse. Total signal counts for animals with multiple detectable bioluminescent signals were added together to determine total tumor burden for a single mouse at a single timepoint. Mice with a saturated signal were excluded from analyses.

### 2.7. Tumor Tissue and Gastrocnemius Muscle Homogenization and mRNA Extraction

Tumor tissue (≤30 mg) was placed into a microcentrifuge tube with beads in ~300 µL (or sufficient volume not exceeding 10% of tissue mass) QIAzol lysis reagent (79306; Qiagen, Germantown, MD, USA). Tissue was disrupted with a bead blaster homogenizer (BeadBlasterTM 24 Microtube, Sigma, St. Louis, MO, USA) with 2 separate rounds of 2–30 s intervals at 619 m/s followed by 1 min of rest. Following lysis, tumor mRNA was extracted utilizing a RNeasy Lipid Tissue Mini Kit (74804; Qiagen, Germantown, MD, USA). Following the addition of chloroform, the upper aqueous phase was removed and placed into a clean tube and washed multiple times. mRNA from homogenized tissue was eluted using RNAse-free water though a RNeasy column.

The left gastrocnemius muscle was homogenized using ≤30 mg of tissue in 300 µL of buffer RLT supplemented with 1% β-mercaptoethanol. Tissue was disrupted with a bead blaster homogenizer (BeadBlasterTM 24 Microtube, Sigma, St. Louis, MO, USA) with 2 separate rounds of 2–30 s intervals at 619 m/s followed by 1 min of rest. Following lysis, mRNA was extracted utilizing an RNeasy Fibrous Tissue kit (74704; Qiagen, Germantown, MD, USA). Proteinase K and RNase-free water were added to each sample, allowed to incubate at 55 °C for 10 min, and centrifuged at 10,000× *g* for 3 min. Supernatant was transferred to a clean tube. Following the addition of ethanol, the upper aqueous phase was removed and placed into a clean tube and washed multiple times. mRNA was eluted using RNAse-free water though an RNeasy column.

The quality and quantity of mRNA was assessed using a NanoDrop 1000. Briefly, 2 µL of RNAse-free water was used to blank the NanoDrop and 2 µL of sample was loaded onto the pedestal and quantified. The quality of mRNA was determined according to the 260/280 and 260/230 ratios.

### 2.8. cDNA and Real-Time PCR

mRNA (1 µg of RNA/reaction) was reverse transcribed to cDNA using Applied Biosystems cDNA synthesis kit (4368814; Fisher Scientific, Suwanee, GA, USA). Real-time polymerase chain reaction (qPRC) was used to evaluate gene expression targets involved in cell cycle regulation, tumor suppression, skeletal muscle mass, metabolism, and inflammation. Regulators of the cell cycle included cyclin D kinase 4 (CDK4) and protein 27 (p27). Tumor suppression targets included protein 21 (p21). F4/80, a macrophage marker, and hairy and enhancer of Split-1 (HES1), a downstream target gene involved in cellular determination and fate, were also included in our analyses. Genes involved in inflammatory responses included F4/80 and tumor necrosis alpha (TNF-α). Phosphatase and tensin homolog (PTEN), an atrophy-associated gene, and peroxisome proliferator-activated receptor-γ coactivator 1 alpha (PGC-1α), a gene involved in skeletal muscle metabolism were also assessed. Table 2 shows all primers used for gene expression analyses. Briefly, Radiant Green HI-ROX SYBR Green was utilized for all qPCR reactions. Glyceraldehyde 3-phosphate (GAPDH) was the housekeeping gene for all qPCR experiments. SYBR green ROX cycling occurred under the following conditions: cDNA was activated at 95 °C for 2 min followed by 20 cycles of 95 °C for 5 s (denaturation) and 60 °C for 20 s (annealing/extension).

### 2.9. Gastrocnemius Tissue Protein Isolation and Quantification

Upon sacrifice, the skeletal muscle tissue was harvested, and muscle weights were taken for the gastrocnemius muscle. Gastrocnemius tissue (≤30 mg) was placed into a microcentrifuge tube with beads in cell lysis buffer (30 µL/mg tissue) containing ice cold radioimmunoprecipitation assay (RIPA) buffer (sc-24948; Santa Cruz, Dallas, TX, USA), supplemented with 10% sodium dodecyl sulfate (SDS), 1% Triton X-100, protease cocktail inhibitor. Tissue was disrupted with a bead blaster homogenizer (BeadBlasterTM 24 Microtube; Sigma, St. Louis, MO, USA) with 2 separate rounds of 2–30 s intervals at 619 m/s followed by 1 min of rest. Samples were placed on ice for 5 min on ice between the 2 separate rounds. Following lysis, protein underwent centrifugation at 10,000× *g* (rcf) for 10 min at 4 °C. Protein supernatant concentrations were quantified using a Pierce BCA protein kit (23225; Thermo Fisher, Allentown, PA, USA).

### 2.10. Western Blotting

Western blotting was used to assess the expression level of proteins regulating skeletal muscle metabolism. Protein samples prepared in 1× loading buffer, supplemented with 10% β-mercaptoethanol, were denatured at 95 °C for 3 min and then immediately placed on ice for 5 min. Protein samples (30 µg/well) were loaded onto 10% SDS-page gels and were run at 225 V for 40 min in 1× running buffer. Following electrophoresis, the gel was placed into 1× Towbin’s transfer buffer, supplemented with 20% methanol, for 15 min. Proteins were transferred onto a 0.45 µm Polyvinylidene difluoride (PVDF-FL) membrane at 100 V for 90 min in 4 °C. Following transfer, membranes were washed once in 1× Tris-buffered saline (TBS) for 5 min. Next, the membrane underwent blocking in Odyssey Blocking Buffer and TBS (1:1) for 1 h at room temp. After blocking, the primary antibodies were added overnight (16 h). Primary antibodies were directed against the following: pAMPK (1:500; CS, #4188), AMPK (1:500; CS, #2532), pSTAT3 Ser 727 (1:500, CS, #9134), STAT3 (1:500; CS, #4904), REDD1 (1:1000; FS, 3PIPA520495), and GAPDH (1:5000; CS, #MAB473). Following removal of the primary antibodies, the membrane underwent 3 × 5 min washes in 1× Tris-buffered saline with Tween 20 (TBST). Secondary antibodies (1:10,0000 in TBST) were targeted to primary antibodies and incubated at room temp for 2 h. Next, membranes were washed twice in 1× TBST and twice in 1× TBS. Membranes were imaged using the Odyssey^®^ Licor CLx System.

Using the Odyssey^®^ Licor CLx System, bands were quantified and expressed using arbitrary units as a measure of integrated optical density. Phosphorylated proteins (pSTAT3) were normalized to total (STAT3) protein expression. Total protein expressions (STAT3, AMPK, REDD1) were normalized to glyceraldehyde 3-phosphate dehydrogenase levels (GAPDH).

### 2.11. Statistical Analyses

An unpaired Student’s *t*-test was used to assess baseline body mass between all control and metformin mice. A mixed-effects model (time × treatment) was use to assess normalized body mass between treatment groups and food consumption for the duration of the study. An unpaired Student’s *t*-test was used to identify any differences in time to caloric intake, signal detection, and length of treatment. Overall survival was determined by a Logrank test. An unpaired Student’s *t*-test was used to compare differences in gene expression, except where variances significantly differed (*p* < 0.05). In those cases, a Welch’s *t*-test was used to compare differences in gene expression between control and treatment animals. Outliers were identified using a Grubb’s test. Significance was established with an a priori alpha value of 0.05. All statistics were completed in GraphPad Prism (Version 9.1, GraphPad Software, San Diego, CA, USA).

## 3. Results

### 3.1. Body Mass in C57BL/6J Mice with NSCLC

**Figure 2:**
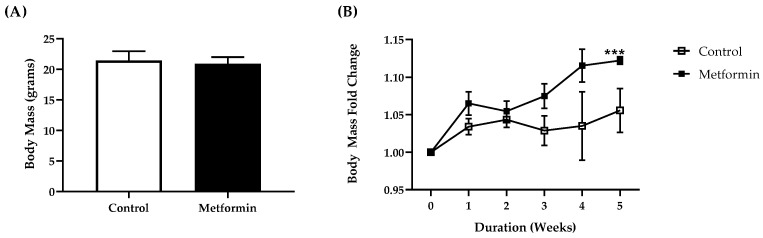
Body weight in C57BL/6J male and female mice following orthotopic LL/2 non-small lung cancer cell implantation. (**A**) Body mass (grams) between control and treatment mice. (**B**) Body mass fold change between control and metformin- (250 mg/kg) treated mice following orthotopic LL/2 cancer cell implantation. Data were analyzed using mixed modeling (time × treatment). *** *p* < 0.001, main effect for time. Control, *n* = 7; metformin, *n* = 9. Data shown as mean ± SEM.

There was no differences in baseline body mass between control and metformin-treated mice (unpaired Student’s *t*-test, *p* = 0.774). As expected, body mass of control and metformin animals increased through the duration of the study (Figure 2). Mixed modeling (time x treatment) from all mice with a detectable bioluminescent signal indicated significant increases in body mass with time [*F*(2.320, 27.85) = 8.788, *p* < 0.001] but not treatment [*F*(1, 14) = 4.510, *p* = 0.0520] or an interaction (time x treatment) effect [*F*(5, 60) = 1.943, *p* = 0.1005]. There were no differences detected between male and female cohorts, supporting the comparison of treatment cohorts pooling both sexes. Since body mass was not significantly different at baseline body mass was represented as a fold change from baseline.

### 3.2. Food Consumption in C57BL/6J Mice with NSCLC

**Figure 3:**
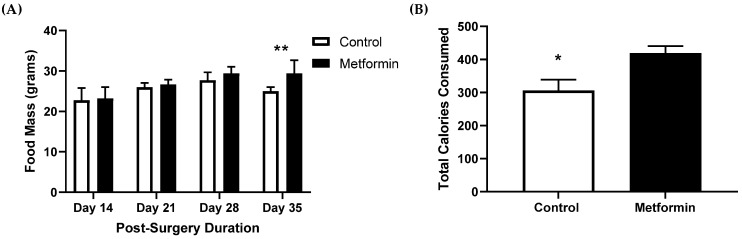
Total food mass and caloric consumption in C57BL/6J male and female mice following orthotopic LL/2 non-small lung cancer cell implantation. (**A**) Food consumption (grams) between control and treatment mice following tumor injection. Data were analyzed using mixed modeling (time × treatment). ** *p* = 0.009, main effect for time compared to Day 14. Control, *n* = 7; metformin, *n* = 8. Data shown as mean ± SEM. (**B**) Caloric Consumption between control and metformin- (250 mg/kg) treated mice following orthotopic LL/2 implantation. Data were analyzed using an unpaired *t*-test. * *p* = 0.018 compared to metformin animals. Control, *n* = 7; metformin, *n* = 8. Data shown as mean ± SEM.

Control and metformin-treated animals continued consuming food for the duration of the study (Figure 3). Overall, most animals maintained a healthy body mass, good ambulatory movement, and an appetite even with tumor burden. Mixed modeling (time × treatment) indicated significant increases in food consumption with time [*F* (2.149, 24.35) = 5.566, *p* = 0.009], independent of treatment. Control mice had significantly lower (*p* = 0.018) total caloric consumption compared to metformin-treated animals (Figure 3).

### 3.3. Time to Tumor Detection and Length of Treatment

**Figure 4:**
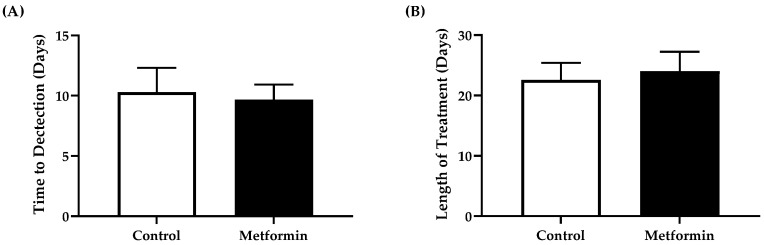
Time to tumor detection and length of treatment in C57BL/6J male and female mice following orthotopic LL/2 non-small lung cancer cell implantation. (**A**) Number of days to a discernable bioluminescent signal following orthotopic injection of LL/2 cells into C57BL/6J mice. Data were analyzed using an unpaired *t*-test. Control, *n* = 7; metformin, *n* = 9. Data shown as mean ± SEM. (**B**) Number of days C57BL/6J mice with NSCLC underwent treatment with control or metformin (250 mg/kg). Data were analyzed using an unpaired *t*-test. Control, *n* = 7; metformin, *n* = 8. Data shown as mean ± SEM.

There was no differences in overall time to detectable bioluminescent signal between control and metformin animals (unpaired Student’s *t*-test, *p* = 0.790) (Figure 4). The treatment timeline between cohorts remained similar, irrespective of treatment (*p* = 0.753) (Figure 4).

### 3.4. NSCLC Tumor Burden and Animal Survival

**Figure 5:**
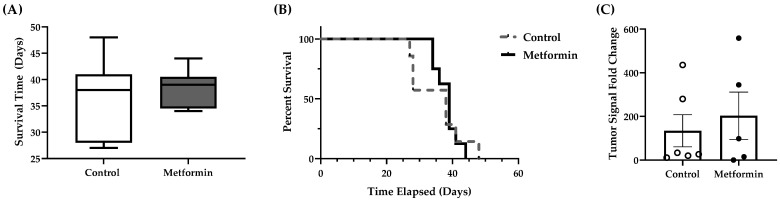

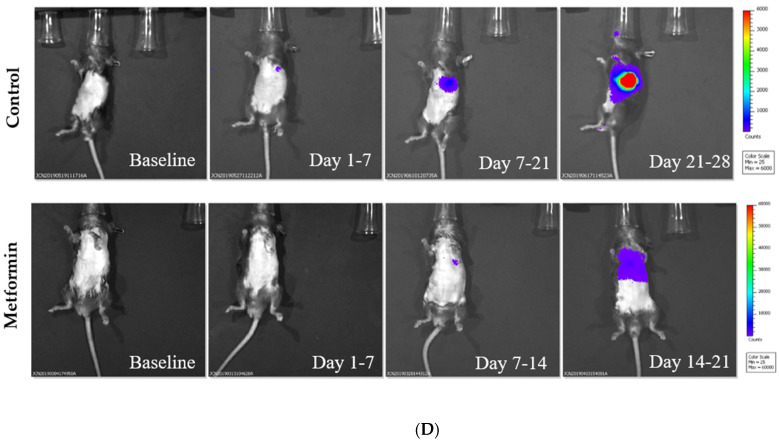
Survival time, tumor burden, and non-small-cell lung tumor growth in C57BL/6J male and female mice following orthotopic LL/2 non-small lung cancer cell implantation. (**A**) Survival duration (days) between control and metformin- (250 mg/kg) treated mice with a detectable bioluminescent signal. Data were analyzed with a Welch’s *t*-test. (**B**) Percent survival of both control and metformin- (250 mg/kg) treated mice following detection of a bioluminescent signal. Data were analyzed with a Log-rank test. Control, *n* = 7; metformin, *n* = 8. (**C**) NSCLC tumor burden fold change between control and metformin- (250 mg/kg) treated mice with a detectable bioluminescent signal. Mice with a saturated signal were removed from analyses. Data were analyzed with a Welch’s *t*-test. Control, *n* = 6; metformin, *n* = 5. Data shown as mean ± SEM. (**D**) Male and female C57BL/6J mice orthotopically administered 1000 Lewis lung carcinoma cells harboring luciferase reporter expression. Bioluminescent signals were tracked throughout the duration of the study via an in vivo imaging system. Once a bioluminescent signal was detected, vehicle or metformin treatment (250 mg/kg, 2× weekly, intraperitoneal injection) began. Top row: Control C57BL/6J female mouse; Bottom row: metformin-treated C57BL/6J male mouse. Scale represents increased tumor burden as signal intensity increases from blue to red.

The mean survival times for control (37 ± 5.6 days) and metformin treatment (40 ± 1.4 days) groups were not statistically significant (Figure 5). There were no significant differences in mean survival time between groups (Welch’s *t*-test, *p* = 0.412). Similarly, no difference was detected in overall survival between control or metformin-treated mice with a detectable bioluminescent signal (log rank test, *p* = 0.827) (Figure 5) nor was there any significant trends (*p* = 0.0515). Some mice developed metastases (control (*n* = 3); metformin (*n* = 3)), which led to an increased tumor burden in those animals. However, mice with a saturated signal were excluded from tumor burden analysis due to limitations within the Living Imaging software (Version 4.5.5, Perkin Elmer, USA). Mice with evidence of metastasis did not exhibit overt indications of declining health compared to mice without metastasis. Moreover, similar tumor burdens were recorded in the groups tested, irrespective of treatment (unpaired Student’s *t*-test, *p* = 0.615) (Figure 5). LL/2 tumor signals representative of observations made in a female control and a male metformin-treated mice were similar (Figure 5).

### 3.5. NSCLC Tumor Gene Expression

**Figure 6:**
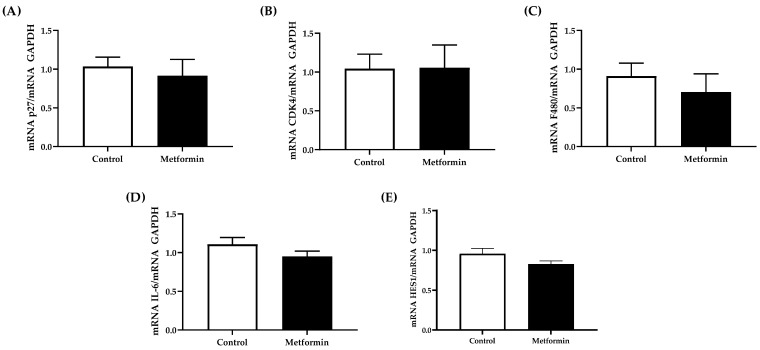
Gene expression in tumor mass collected from C57BL/6J male and female mice following orthotopic LL/2 non-small lung cancer cell implantation. (**A**) mRNA cyclin-dependent kinase inhibitor (p27)/GAPDH; (**B**) mRNA cyclin-dependent kinase (CDK4)/GAPDH; (**C**) mRNA F480/GAPDH; (**D**) mRNA interleukin-6/GAPDH; (**E**) mRNA hairy and enhancer of split 1 (Hes1)/GAPDH. Tumor mRNA expression from C57BL/6J mice with NSCLC concomitant with or without metformin (250 mg/kg) treatment. Data were analyzed using an unpaired *t*-test. Sample size: Control, *n* = 6; metformin, *n* = 6. Data shown as mean ± SEM.

No significant differences were detected in gene expression from NSCLC tumors collected from C57BL/6J mice (Unpaired Student’s *t*-tests, *p* > 0.05). p27, CDK4, F480, IL-6 or Hes1 gene expression were similar between tumors collected from control and metformin-treated mice (p27, *p* = 0.639; CDK4, *p* = 0.973; F480, *p* = 0.488; IL-6, *p* = 0.203; Hes1, *p* = 0.118) (Figure 6). However, Hes1 expression showed a modestly significant effect for sex when males and females are separated within each treatment group [*F*(1, 8) = 6.828; *p* = 0.031].

### 3.6. Maintenance of Skeletal Muscle Mass

Skeletal muscle mass was maintained in all mice, irrespective of treatment (Table 3). Gastrocnemius muscle mass between control (Left: 100.0 ± 7.6 mg; Right: 102.0 ± 8.6 mg) and metformin- (Left: 102.5 ± 6.2 mg; Right: 97.5 ± 6.2 mg) treated mice did not significantly differ (Left: *p* = 0.731; Right: *p* = 0.776).

### 3.7. Gastrocnemius Gene Expression

**Figure 7:**
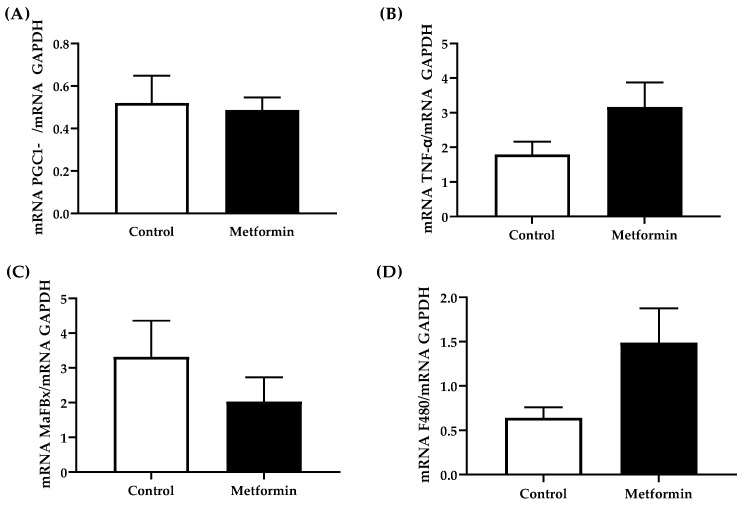
Gene expression in gastrocnemius muscle from C57BL/6J mice with orthotopically implanted LL/2 non-small lung cancer cells. (**A**) mRNA Peroxisome proliferator-activated receptor-gamma coactivator-1alpha (PGC1-α)/GAPDH; (**B**) mRNA tumor necrosis factor-alpha (TNF-α)/GAPDH; (**C**) mRNA muscle atrophy F-box (MAFbx)/GAPDH; (**D**) mRNA F480/GAPDH. Gastrocnemius mRNA expression from C57BL/6J mice with NSCLC concomitant with or without metformin (250 mg/kg) treatment. Data were analyzed using an unpaired *t*-test. Sample size: Control, *n* = 7; metformin, *n* = 7. Data shown as mean ± SEM.

Genes involved in maintaining skeletal muscle mass and inflammatory signaling were not significantly different with regard to treatment (Figure 7). Metformin did not alter skeletal muscle PGC1-α mRNA (*p* = 0.816), MAFBx mRNA levels (*p* = 0.325), TNF-α mRNA levels (*p* = 0.111) or F480 mRNA levels (*p* = 0.076) expression. Two outliers were removed from PGC1-α mRNA expression data. Separation via treatment and sex revealed no significant differences in gene expression.

### 3.8. Gastrocnemius Protein Expression

**Figure 8:**
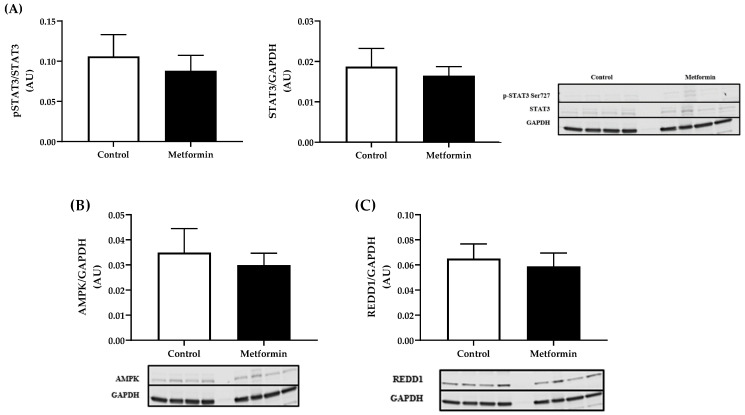
Protein expression in gastrocnemius muscle from C57BL/6J mice with orthotopically implanted with LL/2 non-small lung cancer cells. (**A**) STAT3 expression in gastrocnemius muscle from C57BL/6J mice with NSCLC. Phospho (p)- Signal transducer and activator of transcription 3 (STAT3) Ser727/Total STAT3 and STAT3/GAPDH expression (arbitrary units, AU) in gastrocnemius muscle from C57BL/6J mice with NSCLC concomitant with or without metformin (250 mg/kg) treatment. Data were analyzed using an unpaired *t*-test. (**B**) AMPK expression in gastrocnemius muscle from C57BL/6J mice with NSCLC. Total adenosine monophosphate-activated protein kinase (AMPK)/GAPDH expression (arbitrary units, AU) in gastrocnemius muscle from C57BL/6J mice with NSCLC concomitant with or without metformin (250 mg/kg) treatment. Data were analyzed using an unpaired *t*-test. (**C**) REDD1 expression in gastrocnemius muscle from C57BL/6J mice with NSCLC. Regulated in development and DNA damage responses 1 (REDD1)/GAPDH expression (arbitrary units, AU) in gastrocnemius muscle from C57BL/6J mice with NSCLC concomitant with or without metformin (250 mg/kg) treatment. Data were analyzed using an unpaired *t*-test. Sample size: Control, *n* = 7; metformin, *n* = 8. Data shown as mean ± SEM.

Skeletal muscle proteins that promote atrophy and regulate metabolism did not reveal detectable differences between control and metformin-treated groups (pSTAT3, *p* = 0.5889; STAT3, *p* = 0.6534; AMPK, *p* = 0.6387; REDD1, *p* = 0.6998) (Figure 8).

## 4. Discussion

The present study aimed to assess the effects of metformin as a stand-alone, i.e., monotherapy treatment in altering LL/2 non small lung tumor progression and its ability to support skeletal muscle health during LL/2 tumor progression in C57BL/6J mice. Our data indicate that metformin administered at a dose of 250 mg/kg, twice weekly, via i.p. in an immunocompetent model of NSCLC, was not associated with significant improvements in tumor burden. Moreover, there were no marked differences in gene expression of key tumor cell division (p27, CDK4 and Hes1) and inflammation markers (F4/80 and IL-6) following metformin treatment. Similarly, metformin was not associated with significant improvement in skeletal muscle health. Of note, as no control cohort without LL/2 cells was available, whether skeletal muscles became unhealthy is unknown.

In the conditions tested, no significant differences in tumor fold change or cell cycle regulatory genes (p27 and CDK4) were identified between the control and metformin-treated mice, the possibility exists that our dosing frequency was insufficient to exert effects. Indeed, metformin has a relatively short half-life, a high rate of absorption in the small intestine, and a nearly complete clearance via the kidneys, supporting that in our conditions the bioavailability of metformin is limited and delivery to the tumor site is inadequate [39,40]. Furthermore, it is important to acknowledge that metformin has a hormetic response such that the concentration of metformin within a target tissue influences the mechanism of action, which was elegantly reviewed by Panfoli et al. [41]. Therefore, the tissue concentration supporting the classical effects of metformin as an anti-diabetic drug, may differ from that necessary to alter the cellular and molecular signaling within a tumor. Additionally, appropriate delivery of the anti-cancer therapeutic is of the utmost importance. Oral gavage or via drinking water may prove to be a better route of administration. Indeed, delivering medicines through drinking water results in more consistent drug levels in the plasma when compared to drug delivery via i.p. injections [42,43]. Mice treated with metformin through drinking water, rather than i.p. injections, had an average blood plasma concentration of 32 µM (range of 9.1–55.7 µM), that could allow more consistent drug delivery to the tumor site [42].

Notably, the application of nanoparticle technology has provided an advantageous approach to more innovative cancer treatments. Specifically in NSCLC lines, nanoparticle carriers encapsulated biomolecules and successfully reached target tissues, resulting in either silencing or knockdown of genes to attenuate tumor cell growth [44,45]. Nanocarriers also possess many unique characteristics, making them excellent vehicles for drug delivery with the potential to better regulate pharmacokinetic effects [46]. This could lead to improved uptake of a nanoparticle into a target cell, resulting in increased drug bioavailability such as metformin, more controlled release of a therapeutic, increased drug stability, and reduced side effects from more conventional cancer treatments [47].

Although there was no significant reduction in F4/80 or IL-6 gene expression, animals receiving metformin treatment showed a trend for lower IL-6 gene expression. IL-6 is a multifaceted cytokine that acts as a key mediator of inflammation. High serum concentrations of IL-6 are associated with tumor progression, metastases, and poor clinical outcomes, especially for colorectal cancer patients [48]. In lung cancer patients, metformin has also been shown to reduce IL-6 driven epithelial-mesenchymal transitions, which plays an important role in tumorigenesis [49]. Together these findings suggest that metformin might mitigate tumor migration via effects on IL-6 production.

Metformin has been shown to reduce infiltration of tumor-associated inflammatory macrophages [50]. A previous study indicated that metformin (0.5–2.0 mM in vitro; 100 mg/kg/daily, i.p. in vivo) blocked alternatively activated (M2) macrophage polarization, which is often associated with tumor-driven angiogenesis, tumor migration and invasion, and suppression of anti-tumor immune responses [50]. However, it should be noted that this study differed in terms of metformin dosing strategy administered daily (100 mg/kg, i.p.) versus our twice weekly (250 mg/kg, i.p.). Interestingly, metformin reduced Lewis lung cancer metastases without affecting tumor growth in vivo [50]. This suggests that while metformin may not be directly targeting tumor growth, it is affecting the tumor microenvironment and possibly mitigating metastases. Low-dose metformin (50 mg/kg/day) administration in esophageal squamous cell carcinoma has been previously shown to not affect proliferation or apoptosis of cancer cells, but did increase the formation of tumor-suppressing macrophages in vitro [51]. Similarly, low-dose metformin treatment (250 mg/day) leads to a reprogramming of the tumor immune microenvironment in humans with esophageal cancer [51]. In contrast to the present study, metformin was administered daily, rather than twice weekly, which leads to differing bioavailability of metformin in the tumor microenvironment. As such, metformin may play a significant role in modulation of the tumor microenvironment rather than having a direct anti-tumorigenic impact on the tumor cells, particularly for prostate cancer cells [52].

Key skeletal muscle markers of muscle metabolism (PGC1-α 1) and atrophy (MAFbx) were used to assess overall skeletal muscle health, but exhibited no marked differences in gene expression. However, separating expression based on sex reveals some variation within each treatment, suggesting a potential source of noise evidenced in graphs grouped by treatment (Figure 7). PGC1-α 1 is a transcriptional co-activator critical for regulating energy metabolism and mitochondrial biogenesis [53]. Higher expression of PGC1-α suppresses atrophy-associated genes (muscle RING finger 1 and muscle atrophy F-box (MAFbx)/atrogin-1) and lower expressions of PGC1-α can be associated with rapid muscle atrophy such as cancer cachexia [31]. Metformin has also been previously shown to increase levels of PGC1-α in skeletal muscle via AMPK phosphorylation [30]. Here, neither MAFbx nor PGC1-α showed marked changes during NSCLC cancer development or in response to metformin treatment, suggesting that conditions in this study were not sufficient to induce rapid atrophy (<6 weeks).

In the present study, body mass and gastrocnemius muscle mass were also maintained, indicating that weight loss was probably not an indicative marker of cancer-induced cachexia. Since muscle mass was not significantly affected in this immunocompetent model of LL/2, it is likely that the balance between protein synthesis and degradation was maintained, suggesting that cancer-induced cachexia was not achieved in this study possibly because the endpoint of this study preceded the development of cachexia.

Although there were no significant differences in gene expression markers or correlations between tumor burden or inflammatory markers, a modest inflammatory response occurred within skeletal muscle. Indeed, metformin-treated mice showed a non-significant trend for elevated gene expression levels of markers of inflammation, specifically F4/80, that suggested greater macrophage infiltration and/or activation and the inflammatory cytokine TNF-α. Importantly, infiltration of pro-inflammatory F4/80 positive macrophages has been shown to be linked to obesity, insulin resistance, and cancer cachexia [54,55,56]. Low-grade inflammation coincides with the onset of insulin resistance, which can be indicative of declining skeletal muscle health and reduced glucose disposal. Elevated TNF-α levels are also associated with increased catabolic activity in skeletal muscle, such as protein degradation, insulin resistance, impaired myogenesis and contractile dysfunction [57,58].

Signal transducer and activator of transcription 3 (STAT3), a cytokine transcription factor, has been linked with systemic inflammation in cancer cachexia [59]. Importantly STAT3 is a critical regulator of satellite cell self-renewal and this signaling component plays an important role in muscle wasting, including cachexia [60]. Findings from the present study revealed no phosphorylation of pSTAT3 Ser727 or change in total STAT3 protein expression, suggesting that skeletal muscle wasting, if present, did not occur via this signaling pathway. Because the orthotopic injection mimics tumor development in the lungs, it is possible that a longer timeline or a combination of treatment modalities with irradiation or chemotherapeutics could better mimic the onset of muscle wasting.

Previous studies employing the Lewis lung carcinoma mouse model have shown an attenuation in the expression of fundamental genes involved in the phosphatidylinositol 3-kinase (PI3K)-protein kinase B (Akt) pathway have been observed [61]. The PI3K/AKT pathway, which is often constitutively active in tumor cells, plays an important role in cellular proliferation, growth, metabolism, and protein synthesis [62]. Reduced expression of regulatory genes in the PI3K/AKT pathway could lead to mitochondrial dysfunction and skeletal muscle wasting [61]. Importantly, metformin treatment in tumor bearing rats has been reported to decrease skeletal muscle wasting and improve protein metabolism, attenuating cancer-induced cachexia [63].

Regulated in development and DNA damage response (REDD1) is a ubiquitous protein that is a well-known endogenous inhibitor of the AKT/mTOR pathway [64]. Not surprisingly, this means that REDD1 plays a role in regulating cell growth, mitochondrial function, oxidative stress, and apoptosis [65]. Recent studies have highlighted the importance of REDD1 in maintaining skeletal muscle mass [66]. The present study revealed no differences in REDD1 expression in control or metformin-treated animals. In contrast, a murine model of Lewis lung carcinoma has shown skeletal muscle mass loss between 28–35 days post-tumor development concomitant with increased REDD1 gene expression. The increased REDD1 expression was also associated with lower mTOR expression, suggesting that REDD1 may curb mTOR signaling during later stages of cachexia development [67]. Variations in REDD1 expression in the present study compared to previous investigations [67,68] may be attributed to the variations in lung cancers cell implantation approach.

In vitro incubation of cancer cells with metformin suggested anti-neoplastic potential, although these effects were not supported by our in vivo findings. Futures studies should consider including more frequent metformin dosing in combination with standard chemotherapeutics known to induce deleterious effects to skeletal muscles. In addition, metformin’s potential as a tumor suppressor maybe be supportive in adjuvant therapies or in combination with other cancer treatments. A formative study by Della Corte et al. [69] demonstrated that metformin enhanced the anti-tumor properties of the MEK inhibitor, selumetinib, during in vitro and in vivo treatments. Specifically, the combination of metformin and selumetinib nearly doubled the reduction in proliferation of several human lung cancer cell lines and significantly mitigated tumor growth in mice [69]. Furthermore, human clinical trials demonstrated high safety when combining metformin with erlotinib, a tyrosine kinases inhibitor of the epidermal growth factor receptor, in non-diabetic NSCLC patients as a second-line therapy [70]. Therefore, metformin combination therapies may work synergistically to manage tumor growth by mitigating activity of the PI3K/Akt and MAPK pathways [69,70].

## Figures and Tables

**Table 1:** C57Bl/6J mice survival and metastases following injection with LL/2 cells.

Group	Mice Began Study, *n*	Mice Survived, *n*	Mice with Signal, *n*	Mice with Metastases, *n*
Males	6	3	3	2
Females	6	4	4	1
Total Control	12	7	7	3
Males	6	3	3	0
Females	6	6	6	3
Total Metformin	12	9	9	3

**Table 2:** Primers Used for Gene Expression Analyses.

Primer		Sequence
p27	Forward	TCTCTTCGGCCCGGTCAAT
	Reverse	AAATTCCACTTGCGCTGACTC
F4/80	Forward	CTTTGGCTATGGGCTTCCAGTC
	Reverse	GCAAGGAGGACAGAGTTTATCGTG
CDK4	Forward	ATGGCTGCCACTCGATATGAA
	Reverse	TCCTCCATTAGGAACTCTCACAC
IL-6	Forward	CTGCAAGAGCTTCCATCCAGTT
	Reverse	GAAGTAGGGAAGGCCGTGG
Hes1	Forward	GGTCCTGGAATAGTGCTACCG
	Reverse	CACCGGGGAGGAGGAATTTTT
TNF-α	Forward	CCAGACCCTCACACTCAGATC
	Reverse	CACTTGGTGGTTTGCTACGAC
PGC-1α	Forward	TGATGTGAATGACTTGGATACAGACA
	Reverse	GCTCATTGTTGTACTGGTTGGATATG
MAFbx	Forward	CCAGGATCCGCAGCCCTCCA
	Reverse	ATGCGGCGCGTTGGGAAGAT
GAPDH	Forward	ATGTTTGTGATGGGTGTGAA
	Reverse	ATGCCAAAGTTGTCATGGAT

p27: cyclin-dependent kinase inhibitor protein 27; CDK4: cyclin-dependent kinase 4; IL-6: interleukin 6; Hes1: hairy and enhancer split protein; TNF-α: tumor necrosis factor alpha; PGC-1α: peroxisome proliferator-activated receptor-γ coactivator 1 alpha; MAFbx: muscle-specific ubiquitin ligases muscle atrophy F-box; GAPDH: glyceraldehyde 3-phosphate dehydrogenase.

**Table 3:** Gastrocnemius Muscle Mass in C57BL/6J mice orthotopically implanted with LL/2 non-small lung cancer cells.

	Left Gastrocnemius (mg)	Right Gastrocnemius (mg)
Control	100.0 ± 7.6	102.0 ± 8.6
Metformin	102.5 ± 6.2	97.5 ± 6.2

Data shown as mean ± SEM.

## Data Availability

Data available upon request. Please email jmarin10@uncc.edu for access to data.
